# An analysis of the epitope knowledge related to Mycobacteria

**DOI:** 10.1186/1745-7580-3-10

**Published:** 2007-12-14

**Authors:** Martin J Blythe, Qing Zhang, Kerrie Vaughan, Romulo de Castro, Nima Salimi, Huynh-Hoa Bui, David M Lewinsohn, Joel D Ernst, Bjoern Peters, Alessandro Sette

**Affiliations:** 1 Department of Vaccine Discovery, La Jolla Institute for Allergy and Immunology, 9420 Athena Circle, La Jolla, California, 92037, USA; 2 Portland VA Medical Center/Oregon Heath and Science University, R&D 11, PVAMC, 3710 SW US Veterans Road, Portland, Oregon, 97239, USA; 3 Division of Infectious Diseases, New York University School of Medicine, 550 First Avenue, Smilow 901, New York, 10016, USA

## Abstract

**Background:**

Tuberculosis, caused by the bacterium *Mycobacterium tuberculosis*, remains a leading cause of infectious disease morbidity and mortality, and is responsible for more than 2 million deaths a year. Reports about extremely drug resistant (XDR) strains have further heightened the sense of urgency for the development of novel strategies to prevent and treat TB. Detailed knowledge of the epitopes recognized by immune responses can aid in vaccine and diagnostics development, and provides important tools for basic research. The analysis of epitope data corresponding to *M. tuberculosis *can also identify gaps in our knowledge, and suggest potential areas for further research and discovery. The Immune Epitope Database (IEDB) is compiled mainly from literature sources, and describes a broad array of source organisms, including *M. tuberculosis *and other Mycobacterial species.

**Description:**

A comprehensive analysis of IEDB data regarding the genus Mycobacteria was performed. The distribution of antibody/B cell and T cell epitopes was analyzed in terms of their associated recognition cell type effector function and chemical properties. The various species, strains and proteins which the epitope were derived, were also examined. Additional variables considered were the host in which the epitopes were defined, the specific TB disease state associated with epitope recognition, and the HLA associated with disease susceptibility and endemic regions were also scrutinized. Finally, based on these results, standardized reference datasets of mycobacterial epitopes were generated.

**Conclusion:**

All current TB-related epitope data was cataloged for the first time from the published literature. The resulting inventory of more than a thousand different epitopes should prove a useful tool for the broad scientific community. Knowledge gaps specific to TB epitope data were also identified. In summary, few non-peptidic or post-translationally modified epitopes have been defined. Most importantly epitopes have apparently been defined from only 7% of all ORFs, and the top 30 most frequently studied protein antigens contain 65% of the epitopes, leaving the majority of *M. tuberculosis *genome unexplored. A lack of information related to the specific strains from which epitopes are derived is also evident. Finally, the generation of reference lists of mycobacterial epitopes should also facilitate future vaccine and diagnostic research.

## Background

The goal of the Immune Epitope Database and Analysis Resource (IEDB) [1] is to compile epitope-specific immunological data, as well as analysis tools, and to facilitate the characterization of immune responses in humans and other higher vertebrates. Epitope information can be useful to the scientific community in the design, characterization, and identification of potential vaccines and diagnostics, as well as to assist in basic investigation of immune responses and host-pathogen interactions. In terms of the type of immune responses and associated epitopes considered, we describe epitopes recognized in the context of the adaptive immune response, namely antibody/B and T cell epitopes. Each type of epitope is defined as the molecular structure that is bound by an antibody or T cell receptor.

Curation of data relating to NIAID Category A, B, and C pathogens [2], emerging and re-emerging infectious diseases and various other pathogens into the IEDB is current with the published literature (see [1] for a current list). To date, the database describes epitopes sourced from over 4,000 publications detailing in excess of 32,000 distinct epitopes. Besides epitopes composed of amino acids, the IEDB includes information from all other chemical classes of non-peptidic antigens, including lipids, glycolipids, carbohydrates, DNA, RNA, and small organic molecules. The information can be searched using multiple parameters. For each epitope, specific fields summarize immunological data, including detailed information related to the immunized/infected host organism and source of the antigenic determinant. Associated fields also describe the experimental techniques used to characterize the epitope, and the immunological response detected [3,4]. The IEDB also hosts various bioinformaticstools to analyze epitope data, including: populationcoverage [5]; epitope conservancy and prediction of cellular processing [6]; binding to MHC molecules [7-9];homology mapping of linear antibody epitopes; and 3D structure rendering [10]. The IEDB represents a useful platform upon which comprehensive analyses can be performed for a given host or pathogen of interest.

The mycobacterium *M. tuberculosis *(Mtb) is a highly transmittable human pathogen that causes tuberculosis (TB) and is responsible for more than 2 million deaths globally each year [11]. The uncertain efficacy of the Bacillus Calmette-Guerin (BCG) vaccine for TB in adults, and the emergence of extensively drug resistant Mtb strains have further enhanced the sense of urgency in the development and characterization of TB vaccines and diagnostic reagents [12]. Data specific to the immunology and pathogenesis of TB are helpful in addressing these concerns by facilitating the development and evaluation of new vaccines, therapeutics, and diagnostics, and fostering studies to investigate host-pathogen interactions. Several comprehensive databases provide access to a variety of TB-related information and research sources. These resources include the BioHealthBase Bioinformatics Resource Center [13], The Pasteur Institute TubercuList [14], The Institute for Genomic Research (TIGR) Comprehensive Microbial Resource (CMR) [15], Proteome Database System for Microbial Research at the Max Planck Institute for Infection Biology [16], and the TB Vaccine Testing and Research Materials Contract [16]. However, these databases do not specifically capture/contain immunological data related to TB and associated diseases. The detailed context-dependent data captured within the IEDB therefore provides a unique resource.

Ample data demonstrate the relevance of B and T cell epitopes in reposnse to mycobacterial infections, and for diagnostic and vaccine applications [17-23]. Using the epitope-specific immunological data within the IEDB, a comprehensive analysis wasundertaken to compile and characterize our current knowledge regarding adaptive immunity to the genus Mycobacteria. This analysis was conducted with the following aims: *i*) inventory our current knowledge in the area of mycobacterium-related immunological and epitope data to enhance clinical research and to facilitate the development of vaccines and diagnostic tools; *ii*) identify knowledge gaps from the current literature; *iii*) help identify possible areas for future Mtb-specific research; and *iv*) explore the generation of an reference list of immune epitopes that characterize Mtb, based on input from members of the wider TB scientific community.

## Results and Discussion

### Different types of Mycobacterium epitopes described within the IEDB

Mycobacterial epitopes are described first in terms of their recognition by the humoral and cellular immune response. From a dataset of 1377 unique mycobacterial epitopes (described in the methods section), 1114 are recognized by T cell responses, and 357 by B cell responses. It should be noted that the sum total of the epitopes in the B and T cell categories exceeds the total number of epitopes, as some epitopes are recognized by both cell types. The overall epitope distribution likely reflects the dominance placed on the T cell response in investigating mycobacterial infections, and/or experimental bias.

All but 34 epitopes are peptidic in nature. Of the non-peptidic epitopes, 20 are recognized by B cells and 14 by T cells. Of the B cell epitopes 15 are carbohydrates, 3 are fatty acids, one is a glycolipid, and one is a small organic molecule. Of the non-peptidic T cell epitopes 7 are small organic molecules, and 4 are glycolipids, 2 are carbohydrates, and the last is a lipopeptide. These T cell epitopes are restricted by class Ib/Non-classical MHC molecules. The role of non-peptidic, post translationally-modified, and capsular antigens in Mtb infectivity and pathogenesis, as well as their potential diagnostic use, is well appreciated [24,25]. Thus, this finding identifies a knowledge gap in current epitope data, and highlights the need for the generation of additional data defining non-peptidic and post-translationally modified structures recognized by host immunity against Mtb.

**Figure 1:**
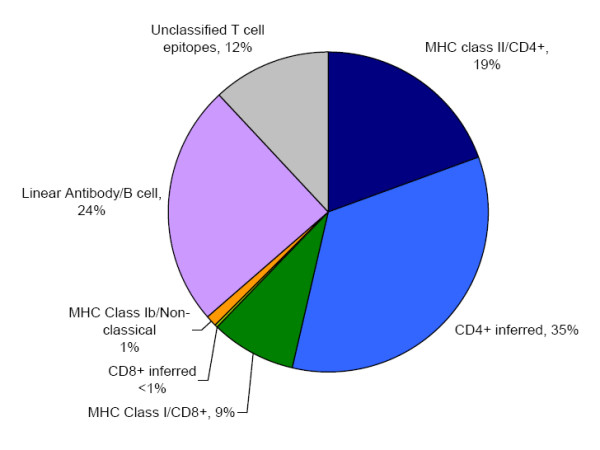
**The nature of antibody/B cell & T cell Mycobacterial epitopes**. The proportion of mycobacterial epitopes according to their type is shown. Where the type of T cell epitope could not be defined directly from the primary information source it was inferred from the assay type classification (described in the methods section).

For this analysis, the assignment of effector cells as CD4^+ ^versus CD8^+ ^T cells was inferred from the assay type classification (described in the methods section) and therefore enabled a larger proportion of epitopes with no reported restriction to be functionally/phenotypically categorized (Figure 1). This categorization showed that the vast majority of epitopes have been defined for CD4^+ ^T cells, and have been described to a much lesser extent for CD8^+ ^T cells. Only a small number of effectors were categorized as non-classical (e.g. recognized by gamma-delta T cells). The dominance of CD4^+ ^epitopes likely represents the focus of the research community in identifying epitopes and antigens restricted by the MHC class II pathway. A significant proportion of the T cell epitopes, mostly described in references utilizing IFNγ and IL-2 assays, remained unclassified due to lack of sufficient information in the original publication regarding MHC restriction and T cell phenotype. By contrast, the classification of B cell epitopes as either linear/continuous or discontinuous was clear-cut No discontinuous epitopes for Mycobacteria have been found. This observation likely reflects the technological difficulties inherent in the definition of discontinuous epitopes, but also highlights a crucial gap in our knowledge of what is recognized during Mtb-specific immune responses.

### Topology and function of the protein sources from which the Mtb epitopes are derived

If we focus specifically on TB, we find that the *M. tuberculosis *genome comprises approximately 4000 protein coding ORFs, for which 924 epitopes are defined in the IEDB from the literature. Most strikingly, these epitopes are derived from only 270 ORFs, corresponding to a mere 7% of the entire genome. In total, as few as 30 ORFs account for 65% of the defined epitopes. Thus, epitope definition studies in Mtb specifically, and mycobacterial species in general, are apparently far from complete. We further analyzed the epitope distribution in the context of the function and topology of the protein of origin.

**Figure 2:**
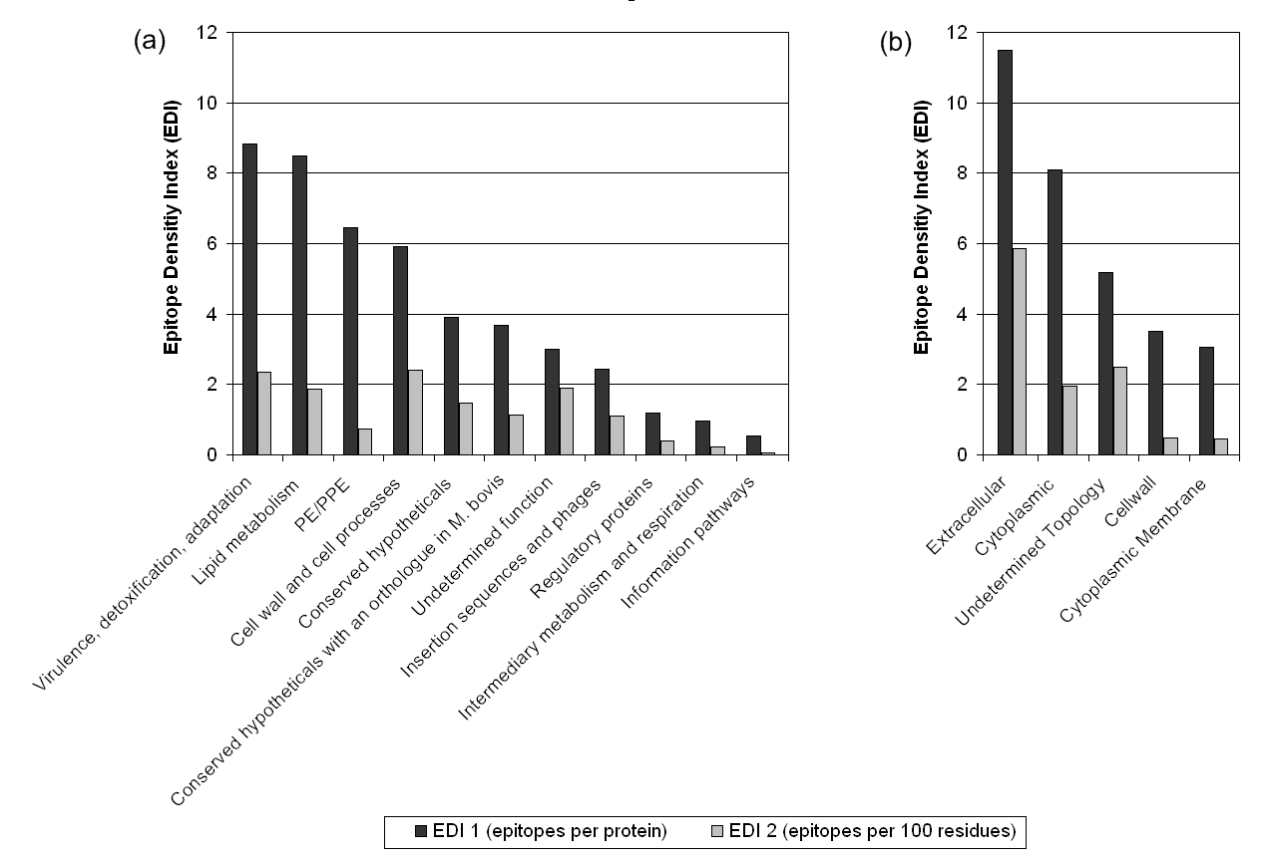
**The epitope density of Mycobacterial proteins**. Epitope Density Index (EDI) values for each protein function (a) and topology (b) category are shown. EDI 1 values (black) represent the number of epitopes in each category divided by the number of proteins with defined epitopes in the category. EDI 2 values (grey) represent the number of epitopes in each category per 100 amino acid residues of proteins with defined epitopes in the category.

In order to explore the distribution of epitopes between proteins according to function and topology category categories (described in the methods section), Epitope Density Index (EDI) values were determined. Two EDI values were calculated for each category. The EDI 1 values represent the number of epitopes in each category divided by the number of proteins (ORFs) with defined epitopes in the category. The EDI 2 values represent the number of epitopes in each category per 100 amino acid residues from the same proteins, allowing protein size to be taken into consideration (Figure 2). In general, epitopes within the IEDB were identified from all functional and topological categories that have defined coding sequences. A small number of epitopes could not be classified, as their source antigen sequences were either not known or not homologous to the species of *M. tuberculosis*, *M. bovis*, *M. leprae*, or *M. avium*.

In terms of protein function, the highest EDI 1 values (8.84 to 5.90) were observed for proteins directly associated with pathogenicity (i.e. virulence, detoxification, adaptation), lipid metabolism, followed by protein classes that are probably exposed on the pathogen surface (i.e. PE/PPE, cell wall and cell processes) (Figure 2a). Fewer epitopes were derived from the organism's internally regulated proteins (i.e. information pathways, intermediary metabolism and respiration, and regulatory proteins), which had an EDI 1 range from 3.90 to 0.54. In general, the EDI values calculated as a function of protein length (EDI 2) correlate with EDI 1 values accept for PE/PEE proteins, which were found to be larger on average. These findings may represent an artifact of the early work directed towards the secreted proteins of Mycobacteria [26-28], while it is also consistent with current knowledge for Mtb pathogenesis; an arsenal of secreted virulence factors and massive lipid metabolism work in concert to establish infection [29,30].

Evidence of mycobacterial proteins changing subcellular location [31], may have an impact on the overall topographical classification of epitopes, and indicates the need to take experimental parameters into greater consideration. With this caveat in mind, the highest EDI 1 values for topology categories are seen in the extracelluar (11.49) and cytoplasmic (8.09) proteins (Figure 2b). Their locations cohobate the findings of the protein function categories. The EDI 1 value for proteins with an undetermined topology was less (5.18), while the lowest values were seen for the cell wall (3.50) and cytoplasmic membrane protein (3.06) categories. The correlation between EDI 1 and EDI 2 values differed for only the cytoplasmic protein category, which have a smaller average size compared to those with an undetermined topology. Finally, the ratio of antibody/B cell and T cell epitopes was equivalent in each protein function and topology category (data not shown).

It is important to note that the above analysis must be viewed with the consideration of biological phenomenon versus experimental bias. It is possible that protein classes described above are highly immunogenic and could represent good candidates for vaccine development. Alternatively, these proteins classes may have received greater attention from the research community, in terms of epitope discovery, because of their high level of expression or ease of isolation. Increased epitope identification in proteins belonging to categories with low epitope density may allow discrimination between these two alternatives, and may also lead to discovery of novel antibody/B and T cell reactivities.

### Mycobacterial strain and species distribution of described epitopes

**Figure 3:**
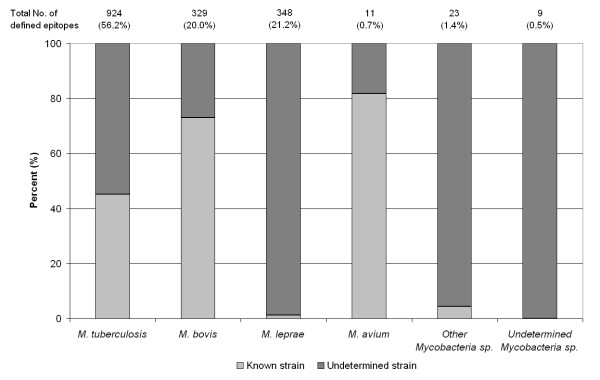
**The distribution of epitopes between Mycobacterial species and strains**. The number of epitopes for each Mycobacteria species considered is presented. The proportion of epitopes with strain information (dark grey), and without (light grey) is shown for each species.

The focus of the present analysis is Mtb, because of its significance as a human pathogen. However, immune epitope data from other all mycobacterium species for which epitopic information is available were also included. These data are important to appreciating the relative balance of knowledge between the various mycobacterium species and their relevance to vaccine and diagnostic target selection. A total of 1644 epitopes have been defined within 11 different mycobacterium species, representing 363 antibody/B cell epitopes and 1281 T cell epitopes. As previously stated, the total of unique mycobacterial epitopes is 1377, therefore some unique epitopes are defined in more than one mycobacterium species or strain, as well being recognized by both B and T cell types [Additional file 1 (sheet1)]. As shown in Figure 3, Mtb epitopes represents 56% of the total, while *M. leprae *(ML) and *M. bovis *(Mb) represent 21 and 20% respectively. Only 11 epitopes have been defined in *M. avium *(>1%), and 23 epitopes are defined in other known mycobacteria species. A further 9 epitopes have been identified in undetermined mycobacterium species.

Evidence that the genetic variability between Mtb strains confers significant phenotypic differences in virulence and immunogenicity [32], underscore the need for strain-specific epitope analyses. Of the 924 epitopes defined in Mtb, 54% were reported with no defined strain information (Figure 3). Of the 417 Mtb strain-specific epitopes, 88% correspond to H37Rv, while the remainder corresponds to strains 103, Erdman, H37Ra and CDC1551 [Additional file 1 (sheet1)]. Of the other Mtb strains of potential interest, such as the laboratory strain H37Ra, only 5 epitopes are known. Additionally, no epitopes are described in other Mtb strains commonly associated with TB, such as the highly virulent Beijing strains, or multi-drug resistant (MDR) and or extremely drug resistant (XDR) strains. A similar picture emerges in the case of the epitopes defined in *M. leprae*, with the majority of the epitopes derived from unidentified strains reflecting the challenges in *M. leprae *strain typing [33,34]. This general lack of strain information for epitopes was found to be, in large part, due to the characterization of epitope recognition following natural Mycobacterial infection in human populations. Indeed, 64% of mycobacterium-specific epitopes within the IEDB were defined in humans. In this context, the identity of the specific strain to which each subject was exposed is often not known and/or not identified diagnostically. Moreover, this omission is compounded by the lack of published sequence information for some of the identified strains considered herein. In the absence of strain characterization information, it is suggested that the geographic location of origin and the ethnicity of study subjects be included in future publications.

### Conservancy and uniqueness of mycobacterial epitopes

Establishing the degree of sequence conservancy for a given epitope among and between mycobacterium species and strains may help differentially identify epitopes applicable to diagnostics, versus vaccine design and evaluations. In the diagnostic setting, epitopes conserved within a species, and not found in any other mycobacterium are of interest [35-37]. For example, reactivity to certain epitopes may distinguish between individuals infected with Mtb and those previously vaccinated with Bacillus Calmette-Guerin (BCG).

A total of 989 mycobacteria epitope sequences can be found in the protein coding ORF's of 3 Mtb strains (described in the methods section), accounting for 72% of all Mycobacterial epitopes [Additional file 1 (sheet 2)]. Of those, 95% are also conserved among Mtb strains. Eleven of the conserved epitope sequences that are also exclusive to Mtb (they do not share >85% sequence identity with any other species) and are also conserved among three Mtb-specific strains [Additional file 1 (sheet 3)]. These epitopes have been identified as potential diagnostic candidates [38,39]. No epitope sequence exclusive to any *M. bovis *strains was reported. Of the 427 epitope sequences found in *M. leprae *TN, 89 are exclusive to this strain. This higher number of unique sequences reflects the greater evolutionary distance between *M. leprae *and the other mycobacterium species considered [40]. Finally, of the 216 epitope sequences that were reported in *M. avium*, 206 were conserved within the two identified *M. avium *strains, though none are exclusive to these strains.

For the purpose of distinguishing between infection with *M. tuberculosis*, infection or sensitization to nontuberculous mycobacteria, or immune responses due to vaccination with BCG, it is essential to know which sequences are not shared with another species, regardless of exclusivity. We found from of a total of 940 epitope sequences conserved between the three Mtb strains, 216 are not present in BCG Pasteur 1173P2 (Table 1). These epitopes might be of diagnostic use in distinguishing between TB infection and vaccination. As expected, a substantially greater number of Mtb conserved epitope are not shared with *M. leprae *and *M. avium*. All of the 955 immune epitope sequences present in the virulent AF2122/97 strain of *M. bovis *are also be found in Mtb, while only 18 of the 940 conserved Mtb epitopes were found to be absent from *M. bovis*.

**Table 1:** The numbers of Mycobacteria B and T cell epitope sequences that are present in one species but absent in another

	The number of epitope sequences present in:				
**Species**	***M. tuberculosis***	***M. bovis***	***M. bovis *BCG**	***M. leprae***	***M. avium***

Epitope sequences not found in:	**940 ^1^**	**955 ^2^**	**761 ^2^**	**427 ^2^**	**206^1^**
***M. tuberculosis***	-	0	8	242	21
***M. bovis***	18	-	8	242	23
***M. bovis *BCG ^1^**	216	202	-	242	23
***M. leprae***	757	770	576	-	53
***M. avium***	748	763	570	271	-

^1 ^The total number of epitope sequences that are conserved between the strains of a species.^2 ^The total number of epitope sequences that are present with the single strain information of a species

Of the 955 sequences present in *M. bovis *strain AF2122/97, 202 epitopes are not found in *M. bovis *BCG Pasteur 1173P2, reflecting known deletions in the BCG genome [41]. These deleted epitopes may be of interest in studying the difference in immune response between the BCG vaccine and virulent *M. bovis*. The majority of the sequences present in *M. bovis *are not found in either *M. leprae *or *M. avium*. A similar pattern is observed in the case of BCG. Of the 427 epitope sequences found in *M. leprae*, 242 are absent in Mtb, *M. bovis*, and *M. bovis *BCG, while 271 are not found in *M. avium*. These sequences may discriminate infections with *M. leprae *and other mycobacterial species. Finally, all of the 206 epitope sequences conserved in *M. avium*, 21 are not present in Mtb, 23 in *M. bovis *and BCG, and 53 in *M. leprae*.

### Hosts range of defined epitopes

In addition to humans, a variety of different host species have been utilized to define mycobacterial epitopes. These other hosts include mice, cattle, rabbits, rats, macaques, chickens, and guinea pigs. As pathogens, mycobacteria are well-established in both human and veterinary medicine. These bacterial infections are of particular significance to the cattle, swine, and fowl industries. *M. bovis *infection in cattle continues to have a substantial financial impact. There is no information available for epitopes recognized in other naturally-infected hosts, such badgers for *M. bovis *or nine-banded armadillos for *M. leprae*.

Accordingly, we have investigated the mycobacterial immune epitope information for the relevant animal models and host species. A total of 1548 host-epitope interactions, defined as an epitope experimentally determined to be recognized within a particular host species, are distributed between different host species and immune cell types. Therefore epitopes within the dataset of 1377 unique epitopes are recognized by more than one host species or immune cell type. The distribution of all mycobacterial epitopes amongst the different host species is shown in Table 2. Not surprisingly, the vast majority of epitopes (63.7%) are defined in human hosts. Murine epitopes account for an additional 24.1%, of which, approximately a third have been defined in HLA transgenic mice, and are therefore restricted by human MHC molecules. The epitopes described in bovine species account for another 7.3% of the total. The number of epitopes identified in other animal models is surprisingly small. For example, only 15 are identified in the rat, and 1 in the guinea pig. In neither case are B cell epitopes represented as they are in the other listed species. Surprisingly, only 9 epitopes have been defined in non-human primates, accounting for less than 1% of the total. Thus identification of additional epitopes in non-murine animal models, specifically those of non-human primates might be of future interest.

**Table 2:** Recognition of Mycobacteria epitopes from different host species

**Host Species**	**No. of B cell Epitope Recognitions**	**No. of T cell Epitope Recognitions**	**Total No. of Epitope Recognitions^1^**
Chicken	2	0	2 (0.1%)
Human	220	813	985 (63.6%)
Rabbit	46	0	46 (3.0%)
Rat	0	15	15 (1.0%)
Mouse	107	290	373 (24.1%)
Macaque	8	1	9 (0.6%)
Bovine	12	101	113 (7.3%)
Guinea pig	0	1	1 (0.1%)
Undetermined	3	1	4 (0.3%)

**Total**	**398**	**1222**	**1548**

^1 ^The total for Both T and B cell epitopes may differ from the sum of epitopes in each category as some epitopes are recognized by both cell types.

In the majority of cases, the number of B cell epitopes identified in each host organism is considerably less than those for T cells, with the exception of epitopes identified in chickens and rabbits. In these species, T cell epitopes have yet to be described. This observation is likely a reflection of the preferential use of these species for antibody production and the study of humoral immunity to TB.

### Epitope recognition in different disease states

Using the definitions established by the CDC (as described methods section), a total of 1460 human disease state classifications are known for the 985 epitopes reported for naturally infected human hosts. As expected, the recognition of some epitopes was reported for more than one disease state, which explains the discrepancy in the above totals. We found that the greatest proportion of human recognitions of immune epitopes (Table 3) are within the Clinically Active TB category (29%), followed by Vaccinated (18%), then Prior TB (11%). Lower numbers are recognized in Exposed but not Diseased [house-hold contacts, PPD-/+, TST-/+] (9%) and TB test positive [TST, PPD, etc.] (7%) groups. *M. leprae *infections account for most of the Exposed to Other Mycobacterium category (23%). Only a small number of epitopes could not be classified under the current disease state scheme (31, not shown); including epitopes recognized in unspecified/unknown or other diseases where mycobacterial involvement is undetermined.

**Table 3:** Summary of epitope recognition in different TB disease states

**Disease State**	**No. of Epitopes Recognized**
**Clinically Active TB**	422 (29%)
**Prior TB**	163 (11%)
**Exposed but not Diseased**	124 (9%)
**Vaccinated**	267 (18%)
**Exposed to other Mycobacterium**	332 (23%)
**Unexposed**	47 (3%)
**TB Test Positive**	105 (7%)

**Total**	**1460**

The IEDB also captures negative immunological data, when it is reported in the literature and found to meet our inclusion criteria. This feature of the database permits a more in depth analysis of immune epitopes differentially associated with certain diseases states. For example, a given investigator might be interested in searching the database for epitopes recognized by individuals with Clinically Active TB, but not by vaccine recipients or individuals Exposed to other Mycobacteria. When we queried the database for this specified type of epitope, 74 different epitopes were found. Similarly, we queried for epitopes reported in individuals in the Exposed/resolved category, but not by those with Clinically Active TB. In this case a total of 27 different epitopes were found. In general however, we noted that for most epitopes, information is sorely lacking regarding recognition in multiple disease states, thus limiting the usefulness of the positive recognition data, especially in diagnostic and prognostic settings. Overall, the analysis underlines the need for controlled systematic studies to correlate immune recognition of particular epitopes and antigens with disease states and outcomes.

### Animal models and protective epitopes

A total 618 mycobacterial epitopes have been identified in non-human hosts. These epitopes been further categorized into TB or non-TB animal models [42]. 492 epitopes have been identified in TB models, while 126 have been identified in non-TB models. As mentioned above, in many instances, these epitopes were recognized in HLA transgenic mice that express human MHC molecules.

Analogous to the human disease outcome scenario, it is useful to be able to identify which of the reported epitopes confer protection in experimental models of the disease. Additional file 1 (sheet 4) lists protective epitopes that have been reported in the literature for animal models of Mtb infection. It should be noted that in our classification of protective epitopes, we utilize a rather stringent definition. Namely, we only consider protective epitopes that are utilized as isolated molecular structures to immunize and confer protection, and do not consider protective epitopes merely associated with protection in contexts were responses directed against multiple epitope specificities are present. Moreover we define protection from disease in these animals as a reduction of clinical signs or reduction in bacterial load.

Utilizing these stringent criteria, only 10 T cell epitopes are shown to be protective in mice and rats. Four of the protective epitopes are derived from the antigen 85 protein, 2 from the 6 kDa early secretory antigenic target (ESAT-6) and 2 from the 60 kDa chaperonin 2 protein. Although a monoclonal antibody specific to a Mtb polysaccharide conferring partial protection on mice has been identified [43], no protective B cell epitopes have been reported in the literature that fulfill the curation requirements of the IEDB. These data indicate that a more comprehensive analysis of the protective nature of TB epitopes is needed.

### MHC binding data of Mycobacterial T cell epitopes

The analysis of MHC binding data can be valuable as a means of preliminary epitope identification, and to confirm restriction assignments. In all, MHC binding information is known for 436 of the 1114 T cell epitopes, of which 148 are bound by more than one MHC molecule. As shown in table 4, the majority of data is related to MHC class II (63%). Class I binding data comprise almost all of the remaining 36%, with Non-classical MHC restricted epitopes (recognized by CD1 and H-2-M3 molecules) contributing approximately 1% of the total.

**Table 4:** The restriction of T cell epitopes by MHC molecules

**Species**	**MHC Class I**		**MHC Class II**		**MHC Class Ib/Non-classical**
	Antigen type	No. of epitopes	Antigen type	No. of epitopes	No. of epitopes

**Human**		**118**		**500**	**4**
	HLA-A	48	HLA-DP	4	-
	HLA-B	61	HLA-DQ	9	-
	HLA-C	0	HLA-DR	449	-
	Undetermined	9	Undetermined	38	-
**Murine**	**-**	**47**	**-**	**113**	**8**
**Rat**	**-**	**5**	**-**	**2**	**0**
**Rhesus**	**-**	**1**	**-**	**3**	**0**
**Chimpanzee**	**-**	**0**	**-**	**2**	**0**
**Bovine**	**-**	**0**	**-**	**4**	**0**

There are 13 different HLA class I antigens that restrict a total of 118 different epitopes [Additional file 1 (sheet 5)]. Epitopes defined for HLA-A and HLA-B antigens are approximately equal, while no mycobacterial epitopes were reported to be restricted by HLA-C antigens. Epitopes bound by HLA-A2, B35, and B53 are the most numerous (46, 28, and 10 restrictions, respectively). Furthermore, 65% of all HLA class I restrictions are determined at the level of allele specificity, with the major alleles being A*0201 and B*3501. There are 500 known epitopes bound by 25 different HLA Class II molecules [Additional file 1 (sheet 5)]. The vast majority of epitopes are bound by DR antigens (90%), while DP and DQ antigens bound less than 10 epitopes each. The greatest number of epitopes are bound to DRB1*0101, *0301, *0401, *1501, and DRB5*0101. Finally, MHC binding information can also identify degenerate MHC ligands, of potential importance for vaccine and diagnostic applications because of their broad population coverage. Here we find 38 mycobacterial MHC ligands that have promiscuous binding properties have been identified [Additional file 1 (sheet 6)].

Next, we wanted to assess whether Mtb epitopes defined in human hosts would allow for balanced coverage of the different HLA class II and class I alleles, especially those most frequently expressed in ethnicities inhabiting areas in which TB is endemic. To this end, the 10 most frequent HLA class I and class II alleles found in each region of high TB incidence were compiled, based on the WHO report on TB incidence [44], and from the dbMHC database [45] of allele frequencies [Additional file 1 (sheet 7)].

Given the lack of breath in HLA allele specific Mtb epitope information discussed above, it is perhaps not surprising that few of these alleles are associated with epitopes of known restriction. Of the HLA class I alleles frequent in endemic areas, 7 are associated with described epitopes. Similarly, of the 33 class II alleles are most frequent in regions with a high incidence of TB, Mtb epitopes are known to be restricted by only 7 of these alleles, which represents just 51 epitopes. There are no epitopes identified for HLA-DP, and only 5 HLA-DQ alleles have been shown to present Mtb epitopes to T cells. Thirty Mtb epitopes are restricted by DRB1*0101, which represents more than half of the allele specific data for HLA-DR. In conclusion, coverage of alleles frequently expressed in endemic areas is sparse, and could be improved by additional research.

HLA antigen associations with the outcome of human TB infection have been reported [46-50]. We have identified 15 HLA class I and 23 class II antigens and alleles antigens [Additional file 1 (sheet 8)], thought to be associated with susceptibility to, or protection from TB, within the published literature. Similarly to what is found in the case of the alleles frequently found in endemic areas, little information exists for the epitopes presented by alleles associated with disease resistance or susceptibility. Interesting results have been obtained in this area in the case of other pathogens such as HIV, for which epitopes restricted by beneficial class I HLA alleles have significantly stronger selective pressure exerted on them [51]. For plasmodium, evidence that HLA class I and II polymorphisms are associated with progression of malaria has also been proposed [52]. In light of this information additional studies in TB might be of interest.

Finally, in terms of non-human hosts, MHC binding data is available for only 5 species: mouse, rat, macaques, chimpanzee, and cattle (Table 3). Murine MHC restriction accounts for roughly 90% of the data. Finally, with regard to specific MHC molecules, only 5 murine MHC molecules have 10 or more known epitopes: H-2-Db, H-2-IAg7, H-2-IAb, H-2-IEg7, H-2-IAd antigens [Additional file 1 (sheet 9)]. In conclusion, significant numbers of MHC restricted epitope data is available for non-human hosts, though the majority corresponds to murine MHC class II molecules.

### Definition of reference set of Mtb epitopes

The IEDB is designed to be as comprehensive and inclusive as possible. No attempt is made to privilege particular datasets based on the origin, experimental technique, or perceived quality or value of the data. This design allows the user to query the database and select particular dataset matching desired characteristics. This approach is rigorous and associated with the least loss of information. However, it is also associated with an important potential drawback, namely the requirement of a fair level of familiarity by the user with the intricacies linked to a particular diseases or experimental systems. For this reason, standardized epitope reference datasets could be of significant use in the assessment of host-pathogen interactions, the development and testing of diagnostic assays, and vaccine evaluations.

To guard against subjectivity and arbitrary inclusion or exclusion of data, we propose that the definition of reference datasets be governed by objective criteria agreed upon by the relevant research community. This strategy has the advantage to allow automatic updates by inclusion of new epitopes that match the objective criteria. We have initially generated epitope reference datasets according to the following criteria: 1) *Ex vivo *detection (defined not needing *in vitro *restimulation for T cell recognition, and by definition, positive binding in all antibody assays) and 2) Use of standardized assays (defined as epitopes associated with a list of assays recognized by community experts as "gold standards"; we tentatively included ICS, ELISPOT, and proliferation for T cells responses; and ELISA and Antigen Competition of Ab Binding for antibody responses). The number of epitopes that fulfill the first two criteria (*ex vivo *and standardized assays) is 735, which represents approximately 53% of the total defined epitopes. (Table 5). These epitopes are describe 344 B cell epitopes and 484 T cell epitopes, of which 93 are recognized by both immune cell types.

**Table 5:** Number of epitopes that satisfy each of the defined criteria for epitope datasets

**Criteria**	No. of Unique Epitopes*	**No. of B cell Epitopes**	**No. of T cell Epitopes**
**All mycobacterial epitopes**	**1377**	**357**	**1114**
Ex vivo^1 ^and standardized assays^2^	735	344	484
Ex vivo and standardized assays + MHC restriction/defined epitope^3^	322	182	186
Ex vivo and standardized assays + generalizability^4^	177	67	166

* The number of unique epitopes does not equal the sum of B and T cell epitopes as some epitopes are recognized by both cell types.^1 ^defined as not needing *in vitro *restimulation for T cell recognition, and positive binding in all antibody assays.^2^defined as epitopes assayed by ICS, ELISPOT, and proliferation for T cells responses, and ELISA and antigen competition of antibody binding for B cell responses. ^3^known MHC restriction and/or defined molecular structures.^4^epitopes have been reported in two different publications or IEDB submissions.

Next, we reasoned that researchers might be interested in different subsets of the epitopes defined by *ex vivo *detection and standardized assays. For example, epitopes with known MHC restriction and/or defined molecular structures. If we apply these criteria, we obtain a reference set of 322 unique epitopes of which 182 B cell epitopes and 186 T cell epitopes (46 epitopes are recognized by both immune cell types) (Table 5, [Additional file 1 (sheet 10)]). It is also of potential interest to define which epitopes have been reported in at least two different publications or IEDB submissions. This limitation defines a reference set of 177 unique epitopes, of which 67 B cell epitopes and 166 T cell epitopes (56 epitopes are recognized by both immune cell types) (Table [Additional file 1 (sheet 11)]). Clinical researchers might also be interested in seeing how the epitopes defined by *ex vivo *analysis and standardized assays are distributed between the TB different disease states, as well as in the more specific sub-category reference datasets. These results demonstrate that multiple customized epitope datasets can be generated to best suite the varied needs of the scientific community. We have also placed a detailed summary of these data on the IEDB website [53] with an accompanying forum discussion thread [54] to obtain research community feedback as to the value of these reference sets, and suggestions on how to improve them.

## Conclusion

A comprehensive analysis of immune epitopes derived from mycobacteria was performed with the aim to provide information that can be used to evaluate different vaccine and diagnostic concepts and design new basic or clinical studies. In total, 1377 epitopes were reported for mycobacteria. The distribution was dominated by *M. tuberculosis*, *M. leprae*, and *M. bovis*, while a handful of epitopes were defined within other determined and undetermined species. The vast majority of epitopes are peptidic in nature, with few non-peptidic or post-translationally modified epitopes identified for mycobacteria. This observation represents a significant gap in our knowledge base, because it is well appreciated that non-peptidic and post-translationally modified antigens are important in Mtb infection and also are of potential diagnostic value.

Roughly three times as many T cell epitopes were identified compared to B cell epitopes. For T cell epitopes, the number of CD4^+ ^T cell epitopes is more than 6 times greater than that for CD8^+ ^T cells. The dominance of CD4^+ ^T cell epitopes likely represents the focus of the research community in identifying epitopes restricted by the MHC class II pathway as mediators of TB immunity, which is due to the established importance of CD4+ T cells in control of *M. tuberculosis *infection in humans, non-human primates, and mice. A small number of non-classical T cell epitopes were also identified. For B cell epitopes, no discontinuous epitope sequences were identified. As an intracellular pathogen, the paradigm has been that antibodies provide a limited role in Mtb immunity. This view has been challenged, with evidence suggesting that B cells modulate the host response and confer optimal containment of the microbe [55], while antibodies can provide protection from infection [43,23]. The role of antibody responses remains relevant to diagnostic applications. This may suggest that more research might be warranted with regard to antibody responses and B cell epitope discovery.

Overall, epitopes are derived from all different protein functional categories of Mycobacteria. However, the majority of epitopes were from proteins directly associated with pathogenicity, those that might be exposed on the surface of the organism, or those that interact with the host. Most strikingly, all of the epitopes reported come from a total of 270 antigens, while the top 30 most frequently recognized protein antigens by B and T cells contain approximately two thirds of the epitopes. This distribution may indicate that these proteins are highly immunogenic or, alternatively, may have received disproportional attention from the research community in terms of epitope discovery. The components of the TB vaccine candidate Mtb72F, which is based upon the fusion of Mtb 32 and 39 kDa protein antigens, are examples of important antigens with little epitope knowledge. The IEDB contains information on two T cell epitopes in the 32 kDa Mtb protein, one of which has also been identified in response to the vaccine candidate [56]. Thus, increased epitope identification in proteins belonging to categories with low epitope density may lead to novel discoveries in Mtb.

For the purpose of identifying epitopes that can be used for diagnostic, therapeutic, and vaccine development, we determined the number of epitope sequences conserved in Mycobacteria species, and the number that are exclusive to a particular strain or species. In general, the lack of sufficiently large numbers of genomic sequences from various strains and isolates hinders a comprehensive comparative study. Epitope sequences conserved within a particular species may prove useful in developing diagnostic candidates for mycobacterial infection, while epitopes exclusive to a mycobacterial species might be of use in investigating host-pathogen interactions and the development of diagnostic tools. For example, differentiating between BCG vaccinated and Mtb infected individuals.

In terms of the host species in which mycobacterial epitopes were identified, almost two thirds were human, followed by mouse and cattle. Very few epitopes (<1%) have been described in non-human primates. This information represents an imbalance of knowledge, and a greater identification of epitopes in non-murine animal models; specifically those of non-human primates would facilitate basic studies, and the quantitative evaluation of new vaccine candidates and diagnostics.

In studying the association of known epitopes with regard to different TB disease states in humans, we found that the greatest number of epitopes is recognized in the Clinically Active TB disease state. The identification of disease state-specific epitopes may represent the first step in the development of new diagnostic and therapeutic reagents for TB. To this end, we have also identified disease state-specific epitopes for animal models of TB, as well as those determined by Exposure to other Mycobacterium, and those for Clinically Active TB. In conclusion the classification of different epitope studies according to different disease states highlights the need for controlled systematic studies to correlate recognition of particular immune epitopes and mycobacterial antigens with disease states and outcomes.

In the interest of investigating the association of HLA expression with Mtb, we have examined the MHC restriction of the reported mycobacterium T cell epitopes. We found that while a large amount of relevant data exists, it appears imbalanced. Of the epitopes with known MHC restriction, approximately 80% are class II, 20%, are class I and less than 1% are non-classical HLA restricted. Most data for class I is described by a small number of HLA antigens and alleles, while almost all of the class II data relates to HLA-DR. The coverage of Mtb epitopes in HLA antigens and alleles that are either found more frequently in TB endemic regions, or are considered to be associated with TB disease in population studies, can both be described as limited. Broader characterization of HLA class I and class II antigens is required in order to understand the connection between HLA restricted epitope repertoires and TB disease, since such information is lacking for most of the known mycobacterial epitopes.

Finally, reference sets of epitope data that could be of significant use to the research community for clinical and basic studies, as well as the development and assessment of diagnostic assays and vaccine evaluations. The uses of a standardized assay and *ex vivo *detection were a prerequisite in determining the suitability of the epitopes considered. These epitopes have been made available on the IEDB website. We expect these multiple reference epitope datasets will be of great utility for the Mtb research community.

In summary, a comprehensive analysis of Mtb and other Mycobacterial B and T cell epitopes has shown that a significant amount of epitope data exists for researchers to utilize. This study provides the general scientific community with an objective evaluation of which information is well represented within the current literature, where gaps exist, and areas that might be addressed by future investigations.

## Methods

### Primary source information

The functionality of Mycobacterial derived B and T cell epitopes that are currently described in the published literature was first determined. As of September 1st 2007, approximately 2000 references containing Mycobacteria-specific keywords were identified by broad queries conducted against the PubMed publication library. Of these, 296 contained epitope information meeting the criteria established for inclusion into the IEDB, as described elsewhere [57]. In addition, epitopes from 2 direct submissions to the IEDB by NIAD-support research contracts were also included (BAA-DAIT-04-39 [58]). It should be noted that only molecular structures of less than 50 residues/5000 Daltons MW are currently captured in the database, and that predicted or modeled epitopes are also not curated.

### Definition of Mycobacterial epitope dataset

All Mycobacterial epitopes within the IEDB were identified by querying database entry fields. A dataset of 1377 unique Mycobacterial epitopes was identified. Information for each unique epitope was utilized to further characterize and separate the dataset as required each analysis described.

To maximize the biological relevance of our analysis we did not classify epitopes based on the presence MHC binding data alone. Also, data in which isolated epitopes were used to both immunize and detect responses were also excluded as these 'cryptic' epitopes may not be representative of the natural immune response. While innate immune responses are also relevant in Mtb infection, it is currently beyond the scope of the IEDB.

### Assignment of CD4^+ ^& CD8^+ ^T cell epitopes

T cell epitopes were further categorized as associated with MHC class I, MHC class II, or non-classical MHC class Ib. The use of purified CD4^+ ^or CD8^+ ^T cells in the assay was also used to classify epitopes as Class I or Class II, respectively. In cases where MHC restriction was not reported an assignment was made based upon the type of used. For CD8^+ ^T cells, assignments were based on chromium release, or cytotoxicity assays. While CD4^+ ^T cells were inferred if the T cell assay measured cell proliferation, DTH, production of lymphokines, such as IL5, IL4, IL10, and TGFβ.

### Assignment of Protein Function and Topology

Proteins for which Mycobacterial epitopes are known were assigned a function and topology category. The 4 protein topology categories defined by the PSORT database [59] and the 12 categories that describe gene function, as defined by the Pasteur Institute [14]. The protein topology catergories are as follows: 1) Extracellular, 2) Cell wall, 3) Cytoplasmic, and 4) Cytoplasmic Membrane. Protein function categories are: 1) Virulence, detoxification, adaptation, 2) Unknown function, 3) Lipid metabolism, 4) Cell wall and cell processes, 5) PE/PPE, 6) Information pathways, 7) Intermediary metabolism and respiration, 8) Insertion sequences and phages, 9) conserved hypotheticals, 10) Conserved hypotheticals with an orthologue in *M. bovis*, 11) Regulatory proteins, and 12) Pseudogenes

### Mycobacterial genomic information

The complete genome sequences from 3 Mtb strains: F11, CDC1551, H37Rv [GenBank: CP000717.1, AE000516.2, AL123456.2]*, M. bovis *strains AF2122/97 [GenBank:BX248333.1] and BCG Pasteur 1173P2 [GenBank:AM408590.1], *M. leprae *TN [GenBank:AL450380.1], and 2 *M. avium *strains: 104, and subsp. paratuberculosis K-10 [GenBank:CP000479.1, AE016958.1] were utilized. The 2 *M. bovis *strains were considered separately so that differences between the BCG vaccine and pathogenic strains could be highlighted. For the species represented by more than one strain, the number of epitope sequences that are conserved was also established. In addition, we determined the number of epitopes exclusive to a given species (if no sequence with ≥ 80% identity is present in other species genomes).

### Disease state assignment of epitopes

Following reported classifications for TB infection in humans (Diagnostic Standards and Classification of Tuberculosis in Adults and Children. 2000) [42] we distinguish Mycobactera epitopes according to: 1) Clinically active TB, 2) Prior TB, 3) Exposed but not diseased 4) Vaccinated, 5) Exposed to other Mycobacterium, 6) Unexposed, and 7) TB test positive.

According to this classification, the Clinically Active TB category includes individuals symptomatic for TB with Mtb present in clinical samples. Both recent transmissions (clustering of cases, close contacts) and remote transmissions are included in this category. The category of Prior TB covers individuals who have had a previous history of TB but are now asymptomatic. Individuals that have abnormal x-ray findings, show no clinical symptoms but are TB test positive are also categorized as Prior TB cases. Individuals in the Exposed but not diseased category had close contacts with diseased individuals but never contracted the disease. The Vaccinated category includes subjects that received *M. bovis *BCG or an antigen derived from BCG or Mtb. Individuals Exposed to Other Mycobacterium such as *M. avium *or *M. leprae*, form a separate disease category, as well as those who test positive for TB but for which no other information is available (presumption of the causative agent is not possible because of PPD cross-reactivity). Unexposed individuals come from non-endemic/low TB burden areas and have not had any contact with TB patients; these individuals are generally TB test negative. Lastly, Animal models of TB include studies in mice, rats, rabbits, guinea pigs, and non-human primates utilizing Mtb, *M. bovis*, *M. bovis *BCG and their derivative antigens, while Non-TB Mycobacterial animal models utilize non-tuberculosis Mycobacterium. Herein, immunization, immunogen, and disease details were used to assign the epitopes to the different categories [Additional file 1 (sheet 12)]).

## Abbreviations

APC: antigen presenting cell; 

HLA: human leukocyte antigen; 

ORF: open reading frame; 

MHC: major histocompatibility complex; 

IL: interleukin; 

TGFβ: transforming growth factor beta; 

TST: tuberculin skin test; 

PPD: purified protein derivative; 

ICS: intracellular cytokine staining; 

ELISPOT: enzyme-linked immunosorbent spot; 

ELISA: enzyme-linked immunosorbent assay; 

DTH: delayed type hypersensitivity assay.

## Competing interests

The author(s) declare that they have no competing interests.

## Authors' contributions

MJB drafted the manuscript with the assistance of all authors. QZ, HHB and MJB wrote the programming code. All authors contributed to the interpretation of the data. AS and BP conceived and oversaw the study. DML and JDE provided field expertise. MJB, QZ, KV, JDC, NS and HHB were responsible for the planning and execution of the analysis. All authors read and approved the final manuscript.

## Supplementary Material

Additional file 1 Additional Tables. These spreadsheets contain detailed information on the results of this analysis as well as large assemblies of epitope information. Click here for file
